# CD146 Expression in Human Breast Cancer Cell Lines Induces Phenotypic and Functional Changes Observed in Epithelial to Mesenchymal Transition

**DOI:** 10.1371/journal.pone.0043752

**Published:** 2012-08-30

**Authors:** Anne-Marie Imbert, Celine Garulli, Elodie Choquet, Myriam Koubi, Michel Aurrand-Lions, Christian Chabannon

**Affiliations:** 1 Institut Paoli-Calmettes, Centre de Ressources Biologiques en Oncologie, Centre de Thérapie Cellulaire, Marseille, France; 2 Inserm, UMR1068, Centre de Recherche en Cancérologie de Marseille (CRCM), Equipe Recherche Clinique, Marseille, France; 3 Université d'Aix-Marseille, Marseille, France; 4 Inserm, UMR1068, Centre de Recherche en Cancérologie de Marseille (CRCM), Equipe Molécules d'Adhésion et Interactions Hôte – Tumeur, Marseille, France; Ghent University, Belgium

## Abstract

**Background:**

Metastasis is an important step in tumor progression leading to a disseminated and often incurable disease. First steps of metastasis include down-regulation of cell adhesion molecules, alteration of cell polarity and reorganization of cytoskeleton, modifications associated with enhanced migratory properties and resistance of tumor cells to anoikis. Such modifications resemble Epithelial to Mesenchymal Transition (EMT). In breast cancer CD146 expression is associated with poor prognosis and enhanced motility.

**Methodology/Principal Findings:**

On 4 different human breast cancer cell lines, we modified CD146 expression either with shRNA technology in CD146 positive cells or with stable transfection of CD146 in negative cells. Modifications in morphology, growth and migration were evaluated. Using Q-RT-PCR, we analyzed the expression of different EMT markers. We demonstrate that high levels of CD146 are associated with loss of cell-cell contacts, expression of EMT markers, increased cell motility and increased resistance to doxorubicin or docetaxel. Experimental modulation of CD146 expression induces changes consistent with the above described characteristics: morphology, motility, growth in anchorage independent conditions and Slug mRNA variations are strictly correlated with CD146 expression. These changes are associated with modifications of ER (estrogen receptor) and Erb receptors and are enhanced by simultaneous and opposite modulation of JAM-A, or exposure to heregulin, an erb-B4 ligand.

**Conclusions:**

CD146 expression is associated with an EMT phenotype. Several molecules are affected by CD146 expression: direct or indirect signaling contributes to EMT by increasing Slug expression. CD146 may also interact with Erb signaling by modifying cell surface expression of ErbB3 and ErbB4 and increased resistance to chemotherapy. Antagonistic effects of JAM-A, a tight junction-associated protein, on CD146 promigratory effects underline the complexity of the adhesion molecules network in tumor cell migration and metastasis.

## Introduction

Metastasis is an important step during the natural history of cancers, as it transforms a local disease into a disseminated and often incurable one. A lot remains to be understood regarding cellular and molecular mechanisms by which tumor cells evade the original site, and re-localize to distant sites. First steps of metastasis include down-regulation of cell adhesion molecules, alteration of cell polarity and reorganization of cytoskeleton. This leads to enhanced migratory properties and resistance of tumor cells to anoikis. Such modifications resemble Epithelial to Mesenchymal Transition (EMT) that occurs in physiological and pathological situations [1]. EMT has been classified into three different subtypes, type 3 being associated with tumorigenesis [2].

CD146 (or MCAM, Mel-CAM, MUC18, S-endo1) was first described on malignant melanomas as a melanoma progression antigen [3]. In normal tissues, CD146 is expressed by smooth muscle cells, placental trophoblasts [4] and a subset of activated T-cells [5]. CD146 is a component of the inter-endothelial junction [6] and is now recognized as a marker of mesenchymal cells [7]. A recent report supports the importance of CD146 as a marker of bone marrow stromal cells with the ability to transfer the hematopoietic microenvironment to heterotopic sites [8].

CD146 is a 113 kDA glycoprotein that belongs to the immunoglobulin superfamily. It contains five immunoglobulin-like domains, one trans-membrane region and a short cytoplasmic tail. The presence of several protein kinase recognition motifs in the cytoplasmic domain suggests the involvement of CD146 in cell signaling [9]. CD146 mediates homotypic and heterotypic cell-cell interactions, although its ligand or counter receptor is not known [10]. Its role in endothelial development is suggested by studies in Zebrafish [11]. Its function in cell migration has been suggested by several observations [12]–[15]. Indeed, forced expression of CD146 in a mouse mammary carcinoma cell line increases its metastatic ability in mouse models [16]. In addition, several reports indicate that CD146 is over-expressed on human prostate cancer cells and that CD146 over-expression increases metastasis of prostate cancer cells in nude mice [17], [18]. Similarly, CD146 expression has been associated with advanced tumor stages in human ovarian cancers and pulmonary adenocarcinomas, predicts early tumor relapse and poor prognosis [19], [20]. We have previously reported that CD146 expression is associated with high grade and triple negative (ER^−^/PR^−^/ERBB2^−^) phenotype in human breast primary tumors and is included in a stromal gene cluster enriched in mesenchymal genes. In addition, we showed that increased risk of death is associated with CD146 expression in the epithelial compartment of breast tumors [13]. These findings have been recently confirmed and extended in a study of 505 primary breast tumor tissues by Zeng et al. [21]. The authors report that CD146 expression is associated with triple-negative breast cancers, high tumor stage and poor prognosis suggesting that CD146 expression might be a potential predictive marker of poor response to treatment. Based on these observations, we investigated whether CD146 expression would induce mesenchymal genes expression in breast carcinoma cell lines. Using four carcinoma cell lines, we show that increased expression of CD146 is associated with loss of cell-cell contacts, enhanced cell migration and increased mesenchymal markers mRNAs expression. Opposite results were found in carcinoma cells with an epithelial-like phenotype. We further show that down-modulation or over-expression of CD146 are associated with opposite changes in JAM-A expression, in heregulin responses and increased resistance to chemotherapy suggesting that poor prognosis of CD146 positive breast tumors is related to CD146-induced EMT and increased resistance to chemotherapy.

## Results

To assess the potential role of CD146 in EMT, different cell lines were used in this study: MCF-7, SKBR3, CAL51 and MDA-MB-231. Levels of CD146 expression and ER, PR and Her2 status are summarized in Table 1. MCF-7 and SKBR3 have been classified in the luminal molecular subtype [22], CAL51 [23] and MDA-MB-231 [22] in the basal subtype. From these wild-type cell lines, we generated MCF-7 and SKBR3 cell lines which expressed high levels of CD146, as well as CAL51 and MDA-MB-231 cells in which expression of CD146 was significantly down-regulated (see Figure S1 for CD146 expression of these modified cell lines).

**Table 1:** Characteristics of the breast cancer cell lines used in this study.

	CD146 sMFI	Her2	ER	PR	Molecular classification
MCF-7	1.12±0.01	−	+	+	Luminal
SKBR3	3.42±0.21	+	−	−	Luminal
CAL51	25.6±4.1	−	−	−	Basal
MDA-MB-231	168+±13	−	−	−	Basal

The specific MFI represents the ratio of the mean fluorescence intensity for the CD146 antibody over the mean fluorescence intensity of the isotypic control (mean ± standard error of the mean of at least six independent experiments).

### CD146 expression is associated with loss of cell-cell contacts

**Figure 1:**
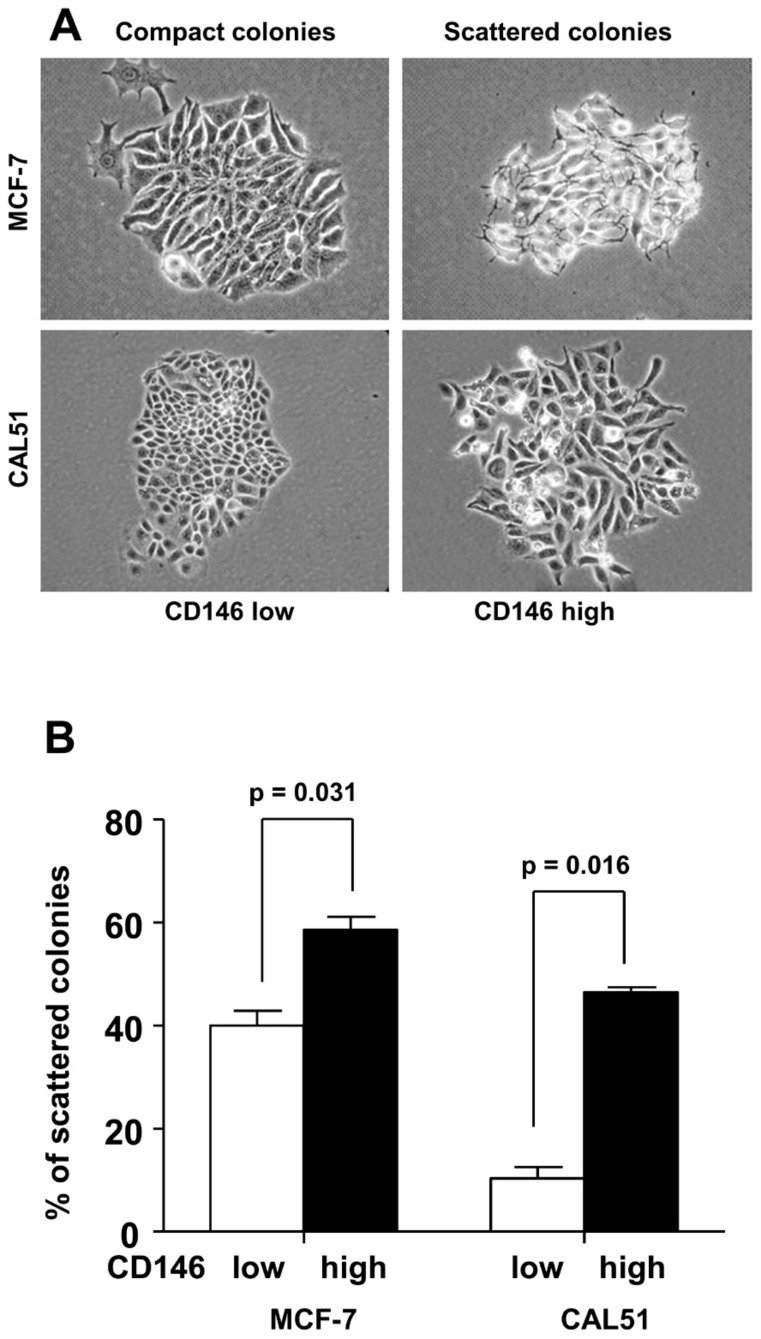
Morphologic changes associated with the modulation of CD146 expression. A: Morphology of MCF-7 cells stably transfected with CD146 and of CAL51 cells stably transfected with a shRNA against CD146 compared to mock transfected cells. The diminution in cell-cell contacts is associated with CD146 expression (magnification×40). B: Proportion of scattered colonies in mocked-transfected and CD146-modulated MCF-7 and CAL51 cells. Cells expressing high levels of CD146 produce a higher number of scattered colonies than their CD146 low or negative counterparts. Results expressed as the mean ± standard error of the mean of at least six independent experiments were analyzed with the Wilcoxon signed rank test.

The morphology of the different cell lines was analyzed in a colony scattering assay. To this end, cells were plated at very low density and the morphology of the colonies was analyzed after 5 days. In these conditions, 60% of mock-transfected MCF-7 colonies were compact colonies (Fig. 1A) with a majority of cells presenting cell-cell contacts. When CD146 was over-expressed, MCF-7 cells were no longer able to form cell-cell junctions, and 60% of colonies were scattered colonies (Fig. 1B). In agreement with these results, the knockdown of CD146 in CAL51 cells decreased the percentage of scattered colonies from 45% to 10%. Quantification of compact versus scattered colonies indicated that the loss of cell-cell contact, a mesenchymal characteristic, was associated with CD146 expression, whereas ability to form cell-cell contacts, an epithelial characteristic, correlated with the lack of CD146 expression (Fig. 1B).

### Modulation of CD146 expression induces changes in mRNA levels for markers of EMT

In order to identify the pathways involved in morphological changes controlled by CD146, we used Q-RT-PCR to measure variations of different EMT marker genes as a consequence of the modulation of CD146 expression. At least six different RNA preparations for each modified cell line were compared to mock transfected cells. We found that CD146 expression in MCF-7 cells was associated with an increased expression of vimentin, N-cadherin and Slug mRNAs (Table 2). Changes in vimentin protein levels were confirmed by immuno-fluorescence analyses (data not shown). Similar results were observed in SKBR3 cells with forced expression of CD146, except for N-cadherin mRNA expression. In CAL51 cells, decreased CD146 expression was associated with increased E-cadherin mRNA expression. Looking for different transcription factors with known role in EMT, only variations of Slug mRNA systematically correlated with CD146 levels in all four cell lines. In SKBR3 and MDA-MB-231 cells, CD146 expression correlated with increased levels of matrix metalloproteinase (MMP)-2 and MMP-9 expression.

**Table 2:** Changes in EMT markers levels are associated with CD146 modulation.

	MCF-7		SKBR3		CAL51		MDA-MB-231	
Vimentin	4.55±0.62	S	2.54±0.34	S	Unchanged		Unchanged	
E-Cadherin	Unchanged		Unchanged		7.03±0.79	S	Unchanged	
N-Cadherin	1.92±0.71	S	Unchanged		Unchanged		Unchanged	
Slug	3.84±0.75	S	3.96±0.72	S	0.32±0.13	S	0.69±0.06	S
Snail	Unchanged		Unchanged		Unchanged		0.49±0.11	S
Twist1	Unchanged		3.26±0.62	S	Unchanged		0.39±0.09	S
MMP2	Not expressed		2.80±0.47	S	Unchanged		0.45±0.06	S
MMP9	Unchanged		2.88±0.46	S	Not expressed		0.43±0.11	S
MCAM	1837±220	S	2025±230	S	0.31±0.09	S	0.19±0.04	S

The CD146-pCMV6-XL4 vector was transfected in MCF-7 and SKBR3 cells, CD146 expression was down-modulated (KD) in CAL51 and MDA-MB-231 cells with siRNAs. mRNAs from CD146 or siRNAs transfected cells were analyzed against mRNAs from mock transfected cells. Results are expressed as the mean of the fold change (± standard error of the mean) for at least six different mRNA preparations. Results were analyzed with the Wilcoxon matched pairs test and considered as significant when p<0.05 (indicated with S for significant).

### CD146 over-expression induces modifications in Estrogen Receptors and response to heregulin

**Figure 2:**
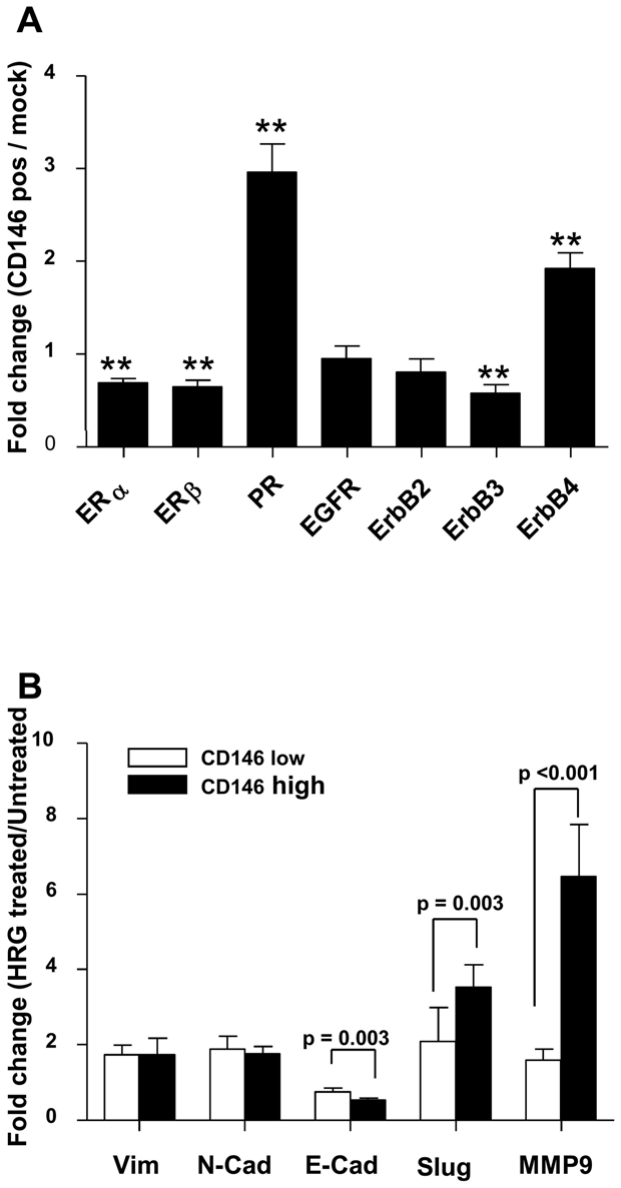
CD146 modulation reveals a link with ER, PR and Erb receptor expression and function. A: analysis of ER, PR and Erb receptors in CD146 transfected MCF-7 cells versus mock transfected cells. Q-RT-PCR was performed on six different RNA preparations. Results expressed as the mean ± standard error to the mean were analyzed with the one sample t-test assuming a theoretical mean equal to 1 (** = p<0.01). B: CD146 increases the response to heregulin in MCF-7 cells. Q-RT-PCR was performed on six different RNA preparations isolated from mock transfected cells (CD146 negative cells) or CD146 transfected cells. Results are expressed as fold change in expression comparing HRG-treated cells with untreated cells for mock transfected cells (CD146 negative) as well as CD146 transfected cells. Mean ± standard error to the mean is shown. Results were analyzed with the Wilcoxon signed rank test.

Having previously shown that CD146^+^ breast tumors are mainly ER^−^PR^−^ and that there is no correlation between the CD146 expression and the ErbB2 status [13], we quantified the transcripts encoding ER (α and β), PR and Erb receptors in MCF-7 cells over-expressing CD146 as compared to control MCF-7 cells (Fig. 2A). We found that CD146 over-expression reduced expression of ERα, ERβ and Her3 receptors, whereas PR and Her4 were up-regulated.

Since forced CD146 expression in MCF-7 cells induces changes in Erb receptors, we tested whether CD146 expression would affect the response to heregulin. We observed a two fold increase in vimentin and N-cadherin expression when mock-transfected MCF-7 cells were cultured with heregulin while E-cadherin was decreased by 20%. When CD146^+^ MCF-7 cells were similarly exposed to heregulin, variations in vimentin and N-cadherin expression remained unchanged, but E-cadherin expression was decreased by 40%, suggesting that the EMT-promoting effect of CD146 is augmented by heregulin (Fig. 2B). This effect on E-cadherin expression was accompanied by up-regulation in transcripts encoding Slug and MMP-9.

### Modulation of CD146 expression is associated with changes in breast tumor cells behavior

**Figure 3:**
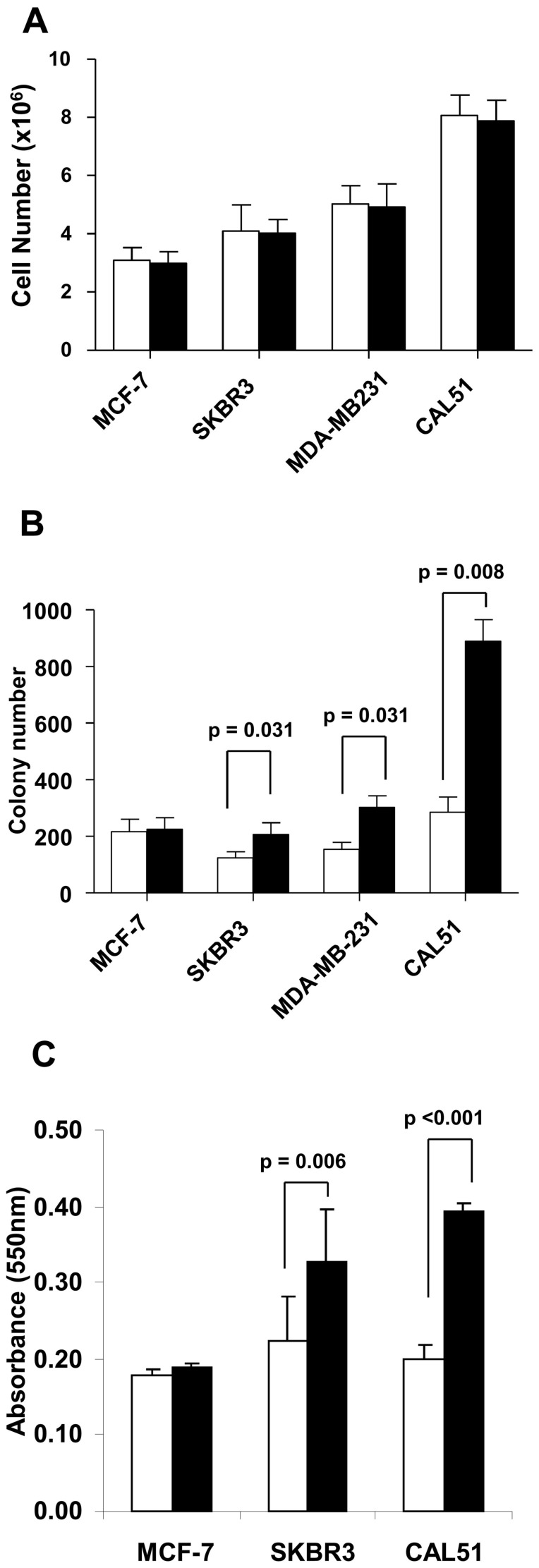
Functional properties of breast cancer cells depend on the level of CD146 expression. A: Anchorage-dependent growth was estimated by the cell number after four days of culture. B: Anchorage-independent growth was assessed by soft agar colony-forming assays as described in Materials and Methods. C, Chemotactic migration evaluated after 36 hours (CAL51 cells) or 96 hours (MCF-7 and SKBR3 cells) using uncoated Boyden chambers and 10% FCS as chemo-attractant. Migrating cells were stained with Crystal Violet solution, lysed and absorbance measured at 550 nm. Results represented as the mean ± standard error of seven independent experiments were analyzed with the Wilcoxon signed rank test. White bars: CD146^low^ cells, black bars: CD146^high^ cells.

To evaluate whether CD146 expression affected cellular proliferation we evaluated the proliferation rate in anchorage-dependent and anchorage-independent growth assays. CD146 expression did not modify the expansion rate of the four cell lines in anchorage-dependent conditions (Fig. 3A). These results were confirmed by studies on cell cycle (data not shown), in which no difference could be detected as a consequence of CD146 modulation. In contrast, we found that reduced CD146 expression in MDA-MB-231 and CAL51 cell lines inhibited anchorage-independent growth rate, while over-expression of CD146 in SKBR3 cells increased its anchorage-independent growth ability (Fig. 3B), suggesting that CD146 contributes to tumor transformation in several breast carcinoma cell lines except MCF-7.

To further explore whether CD146 expression also affects tumor dissemination, we tested the migratory properties of the cell lines manipulated for CD146 expression in Boyden chambers assays (Fig. 3C). The fraction of cells that had migrated was evaluated by colorimetric measurement in triplicate experiments, a semi-automated technique providing more reliable and robust results than manual cell counting (data not shown), provided that sufficient numbers of cells have moved through the filter; this can be achieved only with a later readout (30 hours for the MDA-MB-231 cell line and 96 hours with the slowly migrating MCF-7 cell line) than in most published reports. CD146 down-modulation reduced the migration of CAL51 cells by 47%±3% (when compared to mock-transfected cells) whereas overexpression of CD146 in SKBR3 cells increased migration by 58%±18%. We were unable to demonstrate any change when working with the MCF-7 cell line, suggesting that CD146 forced expression in MCF-7 cells did not recapitulate the full spectrum of EMT-related changes, possibly due to either antagonizing effects of other molecules, or to partners missing for full signaling in a CD146 dependent pathway.

Finally, we studied the chemosensitivity to docetaxel and doxorubicin according to the level of CD146 expression. Cells were exposed to varying concentrations of docetaxel (0 to 100 nM) or doxorubicin (0 to 10 µM) for 72 hours (Table 3), two agents that are widely used as chemotherapy for breast cancer. CD146^high^ MCF7 cells exhibited enhanced resistance to both doxorubicin (+34%) and docetaxel (+57%). CD146^high^ MDA-MB-231 cells exhibited enhanced resistance to doxorubicin only (+70%). Similar results (resistance correlated to the level of CD146 expression) were obtained for SKBR3 and CAL51 cell lines (data not shown).

**Table 3:** IC50 values for doxorubicin and docetaxel depending on the level of CD146 expression.

		CD146 sMFI	IC50 (nM)	P value
MCF-7	Doxorubicin	1.12±0.01	396±86	0.031
		536±42	533±92	
	Docetaxel	1.12±0.01	1.51±0.46	0.024
		536±42	2.37±0.62	
MDA-MB-231	Doxorubicin	15.4±1.0	0.72±0.19	0.036
		168±13	1.23±0.34	
	Docetaxel	15.4±1.0	1.94±0.73	0.562
		168±13	2.03±0.63	

The specific MFI represents the ratio of the mean fluorescence intensity for the CD146 antibody over the mean fluorescence intensity of the isotypic control (mean ± standard error of the mean of at least six independent experiments). IC50 is expressed as the mean (± standard error of the mean) for six different experiments. Results were analyzed with the Wilcoxon matched pairs test and considered as significant when p<0.05.

### Opposite variations in CD146 and Jam-A expression

**Figure 4:**
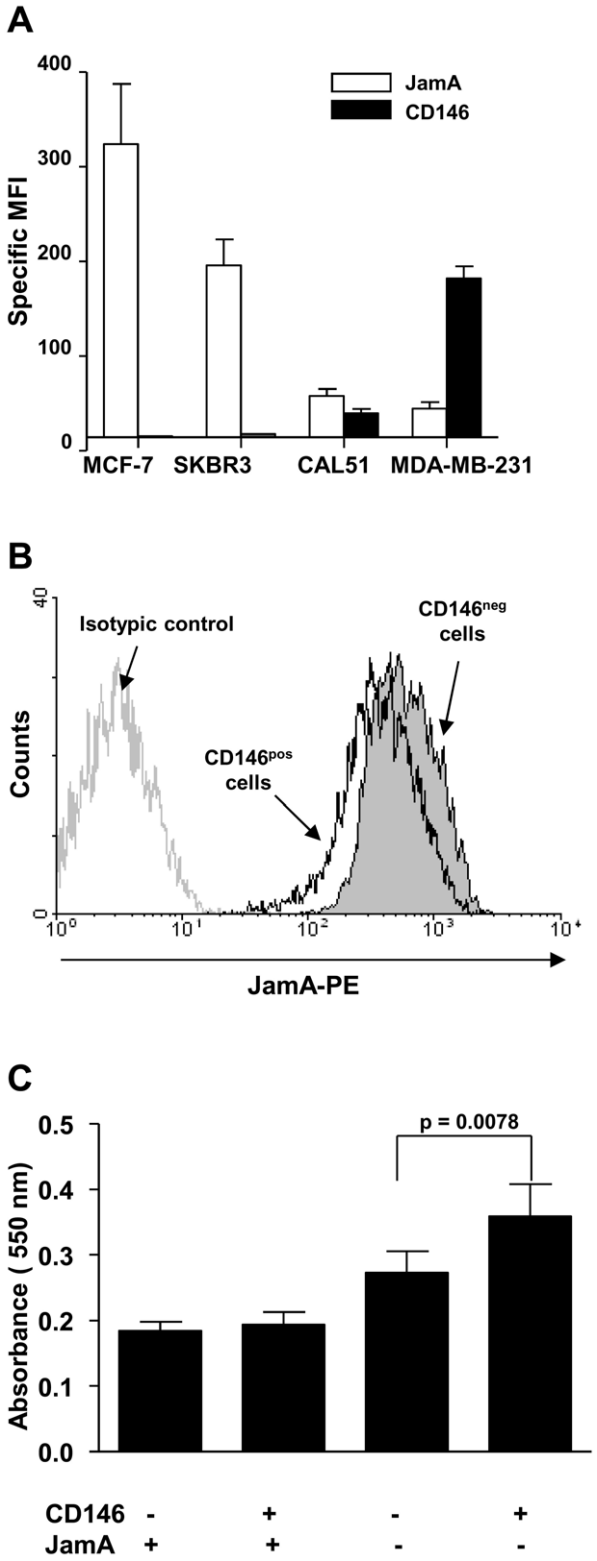
JAM-A expression and migration abilities of breast cancer cells. A: Mean Fluorescence Intensities (MFI) of CD146 and JAM-A expression on indicated human breast cancer cell lines are shown. Values indicate the specific mean fluorescence intensity (sMFI) ± the standard error of the mean of at least seven experiments. The sMFI was defined as the ratio of the MFI for the considered antibody over the MFI obtained with the appropriate isotypic control. B: down-regulation of JAM-A expression by over-expression of CD146 in MCF-7 cells. JAM-A expression measured by flow cytometry in CD146^+^ MCF-7 cells, one representative experiment. C, migration of mock-transfected or CD146^+^ MCF-7 cells after JAM-A knockdown. JAM-A was inhibited with a siRNA in mock transfected and CD146^+^ MCF-7 cells. Results represent the mean ± standard error of the mean of three independent experiments.

Since expression of the Ig superfamily adhesion molecule JAM-A has been controversially associated with the pro-migratory properties of breast cancer cells through integrin activity regulation [24]–[27], we searched whether CD146 and JAM-A variations in expression revealed a consistent pattern. We observed in the four cell lines that JAM-A expression was inversely related to CD146 expression (Fig. 4A). Furthermore, forced expression of CD146 in MCF-7 cells induced a statistically significant decrease in JAM-A expression (23.3%±2.5%, p = 0.0313) (Fig. 4B).

As MCF-7 cells express particularly high levels of JAM-A, we hypothesized that the high level of JAM-A in MCF-7 cells may prevent the possible increase in the migration induced by CD146 expression. We therefore down-modulated JAM-A expression by transient transfection with three different commercial siRNAs. One of those reduced JAM-A expression at the cell surface by 94%±1% (see Figure S2 for JAM-A expression in siRNA transfected cells). Similarly to previous observations with T47D cells [26], we found that JAM-A inhibition alone induced an increase in the migration of MCF-7 cells (Fig. 4C). When the same siRNA was transfected into MCF-7 cells overexpressing CD146, the migration was increased by 30% when compared to JAM-A inhibition alone, suggesting that high levels of JAM-A prevent the acquisition of migratory abilities by MCF-7 cells when these cells are forced to express CD146.

## Discussion

EMT is a reversible differentiation process by which epithelial cells lose their characteristics to acquire mesenchymal properties. EMT occurs in a physiologic way during development, and contributes to tissue repair. EMT also provides neoplastic cells with migratory and invasive properties, and this may be one mechanism by which they leave the primary epithelial tumor site and establish metastases [1], [2]. The first step of EMT involves the loss of intercellular junctions (tight junctions, adherens junctions and desmosomes). Overexpression of transcription factors including Snail, ZEB or members of the bHLH family suppresses epithelial markers and induces the expression of mesenchymal genes leading to cytoskeletal changes and increased motility and migration [28]–[30].

Here, we show that CD146^+^ cells loose cell-cell contacts. CD146^+^ cells also have increased anchorage-independent growth and migratory abilities when compared to their CD146^−^ counterparts. These results are consistent with recently published observations [14], [15], [21], except for MCF-7 cells which express JAM-A at high levels. We found that forced expression of CD146 in MCF-7 cells did not by itself modify their migration ability in contrast to other cell lines, although it induced some of the molecular and cellular changes associated with EMT. When combining JAM-A inhibition and CD146 overexpression, migration of MCF-7 cells was increased when compared to JAM-A positive cells without CD146 expression. Our results suggest that CD146 and JAM-A exert opposite effects, and that inverse modulation of both molecules increases the amplitude of EMT-related changes in MCF-7 cells. A possible mechanism involves the PI3K-Akt pathway as it has been shown both that CD146 activates the PI3K-AKT pathway in melanoma cells and that reduced expression of JAM-A increases the pool of available PIP3 [31], [32]; one could hypothesize that JAM-A blocked the pro-migratory function of CD146 by trapping PIP3 and that high expression of JAM-A in MCF-7 cells prevents part of CD146 signaling via PI3K-Akt. Our observation supports the hypothesis that a complex network of adhesion molecules contributes to tumor cell migration and metastasis with variations in different cell contexts since a similar mechanism was not active in SKBR3 cells.

Together with loss of cell-cell contacts, increased anchorage-independent growth and migratory abilities, CD146 expression is associated with an increase in Vimentin and N-cadherin expression. These results support a role for CD146 in inducing EMT in breast cancer cells. Q-RT-PCR analyses of different transcription factors in four breast cancer cell lines showed that some but not all of classical EMT makers varied in relation to changes in CD146 expression; notably, the variation of Slug expression was correlated with the level of CD146 expression at the cell surface. It was first described that Slug transfection in NBT-II cells (bladder carcinoma) induces the first step of EMT to an intermediate stage characterized by modulation of cell-cell adhesion [30]. This intermediate level of EMT could explain that changes in EMT markers observed in this study are not identical for all cell lines; alternatively modulation of CD146 may be insufficient to recapitulate the full spectrum of EMT, because CD146 acts in the context of a complex signaling network, as suggested by the above discussed interaction with JAM-A.

In a genome-wide transcriptional profiling of human breast cancer cell lines, Blick et al. [33] showed that Slug is highly expressed in basal B cell lines that also over-express vimentin, N-cadherin and fibronectin, whereas E-cadherin is down-regulated. In primary tumors, Slug expression is also associated with the basal-like phenotype [34]. We thus suggest that CD146 participates in EMT by increasing Slug expression, although we cannot conclude on the direct or indirect relation between these two molecules. Recently, in addition to increased expression of SLUG, Zeng et al. [21] suggested that CD146 induced EMT is associated with the activation of the small GTPase RhoA.

Our results also indicate that the induction of CD146 expression in MCF-7 cells induced a slight but significant down-regulation of ERα (30% when compared to the mock-transfected cell line). The knockdown of ERα results in an increase in Slug mRNA [35] while the activation of ERα induces the down-modulation of Slug expression either by a direct association with the Slug promoter or by inhibiting the GSK-3β activity [36]; analyses of 500 breast tumors demonstrated a strong inverse correlation between Slug and ERα expression. In primary breast tumors, CD146 expression is associated with ERα negative tumors [13]. Thus, it is possible that CD146 modulates Slug expression through ERα signaling and not directly.

Heregulin, a ligand of HER receptors and more specifically of ErbB3 and ErbB4, which is essential for ErbB2 phosphorylation in response to heregulin [37], is involved in the progression of different types of human cancers [38] and induces the spread of breast cancer cells in vivo [39]. Heregulin has been shown to induce EMT [40] on SKBR3 cells. Our results indicate that CD146 expression enhanced the response to heregulin, in terms of Slug, MMP2 (increased) and E-Cadherin (decreased) expression. This response was associated with the modulation of Erb receptors in CD146 overexpressing cells and more specifically with an increase in ErbB4 expression.

Cytotoxic assays indicate that the increase in the level of CD146 expression is associated with an increased resistance to doxorubicin and docetaxel (except for MDA-MB-231 cells and docetaxel, maybe due to the residual expression of CD146 in the shRNA-transfected cells). Mostert et al. [41] have demonstrated that in the normal-like breast cancer subtype (10% of breast cancer) circulating tumor cells (CTC) are CD146 positive and then potentially more resistant to chemotherapy.

We conclude that CD146 expression is associated with a mesenchymal morphology and phenotype, the modulation of EMT markers and an increase in cell motility. The increase of the anchorage-independent growth mediated by CD146 suggests an explanation for the higher tumorigenicity of CD146^+^ cell lines. In a precedent work [13] we demonstrated that CD146 expression was associated with poor prognosis in primary breast cancers. Here, we show that CD146 directly or indirectly contributes to EMT *in vitro* which may explain higher metastatic potential and poor prognosis observed in patients with tumors over-expressing CD146. CD146 interacts with Erb signaling by modifying cell surface expression of ErbB3 and ErbB4 and enhancing the response to heregulin. In addition to its relation with EMT, CD146 expression is also associated with increased chemoresistance. All these observations are consistent with CD146 expression on malignant breast cells tumors being an adverse prognosis criterion.

## Materials and Methods

### Cell lines

Four breast tumor cell lines (Table 1) were used in this study: MCF-7, MDA-MB-231, SKBR3 (all from American Type Culture Collection, Manassas, VA, USA), and CAL51 [42]. All cell lines were cultured in RPMI (Lonza, Basel, Switzerland) supplemented with 10% heat-inactivated fetal calf serum (FCS, Invitrogen, Paisley, UK). For MCF-7 cells the same medium was supplemented with insulin (30 µg/mL, Sigma-Aldrich, St Louis, MO, USA). All culture media contained 100 U/mL penicillin and 100 µg/mL streptomycin (Invitrogen).

### Stable modulation of CD146 expression

29mer Short Hairpin RNA (shRNAs) directed against CD146 were obtained from Origene Technologies, Inc. (Rockville, MD, USA). CD146 cDNA as transfection-ready DNA was obtained from Origene Technologies, Inc. (Rockville, MD, USA).

shRNA expression vectors in pRS plasmid and the CD146 expression vector in pCMV6-XL4 plasmid were amplified and purified with Nucleobond PC 100 Kit (Macherey-Nagel GmBH & Co, Düren, Germany).

Cells were plated at 3×10^5^ cells in six-well plates. Transfections were performed using Fugene-6 (Roche Diagnostics, Meylan, France) as directed by the manufacturer. 48 hours after transfection, puromycin (0.8 µg/mL, Sigma-Aldrich) was added. Transfected cell lines were grown in presence of puromycin.

The negative controls for CD146 expression are the cell lines transfected with the pCMV6-XL4 vector (overexpression) or the TR20003 vector (knockdown).

### RNA-interference mediated JAM-A silencing

siRNA duplexes directed against JAM-A were obtained from Sigma-Aldrich. A siRNA that recognizes the green fluorescent protein gene was used as control.

10^5^ cells were plated in six-well culture dishes in 2.5 mL medium without antibiotics. Cells were transfected with a mixture of siRNA (10 nM) and Lipofectamine RNai/Max (Invitrogen) according to the manufacturer's protocol.

### Quantitative real-time polymerase chain reaction (Q-RT-PCR)

Total RNA was isolated from 1 to 2×10^6^ cells using an RNA extraction kit (Macherey-Nagel), denatured at 65°C for 10 min and reverse transcribed using Superscript II reverse transcriptase (Invitrogen). Q-RT-PCR was tested using SYBR Green reagent on an ABI7700 system (Applied Biosystems, Foster City, CA, USA). Specific primers (Table S1) were designed using the Primer Express Software (Applied Biosystems). Gene expression was normalized for RNA concentration with β-Actin. The relative level of expression for a particular gene was evaluated using the 2^−ΔΔCt^ function [43].

### Flow Cytometry

Analyses were conducted with a LSRII Flow cytometer (Becton-Dickinson Immunocytometry Systems, San Jose, CA, USA). The antibodies used in this study were as follow: PE-conjugated anti-CD146 (BioCytex, Marseilles, France) and Alexa Fluor® 647-conjugated anti-CD321 (JamA-F11R, Becton-Dickinson, San Diego, CA). Cells were incubated with antibodies for 30 minutes on ice. Isotype controls were used to set up the threshold for positivity. Dead cells were gated out by staining with Dapi (1 µg/mL, Invitrogen). The specific mean fluorescence intensity (sMFI) was defined as the ratio for the considered antibody over the mean fluorescence intensity obtained with the appropriate isotypic control.

### Colony scattering assay

Five thousand cells were plated in 100 mm plate. After 5 days, 200 colonies were analyzed for their morphology. In scattered colonies, less than 75% of the cells exhibit cell-cell contacts. Other colonies are considered as compact colonies. Six independent plates were analyzed [44].

### Anchorage dependent and independent growth assay

To evaluate anchorage dependent growth, 10^6^ cells were plated in 100 mm-culture dishes and cells were quantified at day 4.

For anchorage independent growth assay, cells were suspended in 1 mL of 0.3% agarose (Caltag Laboratories, Burlingame, CA) in RPMI supplemented with 10% FCS and plated in triplicate in six-well plates on 2 mL of pre-solidified 0.5% agarose in the same medium, with 1 mL of medium covering the cells. For MCF-7 cells, medium was supplemented with insulin (30 µg/mL). The cells were incubated at 37°C in 5% CO_2_. Colonies were counted after 3 weeks. Seven experiments were analyzed (except for MCF-7, 4 experiments).

### Cell migration assays

Before migration, cells were starved overnight (6 hours only for MCF-7 cells) in RPMI medium (Lonza) supplemented with 0.1% Bovine Serum Albumin (BSA, Sigma). Migration was observed in transwell culture inserts of 6.5 mm diameter and 8 µm pore filters (Greiner Bio-One SAS, Courtaboeuf, France). 3×10^4^ cells in 100 µL of RPMI medium with 0.1% BSA were seeded in the upper compartment and 600 µL of RPMI 10% FCS were added to the lower chamber. Cells were allowed to migrate for 30 h at 37°C for CAL51 cells and 96 h for MCF-7 or SKBR3 cells. After removing cells on the upper side of the transwell, cells on the underside were stained with 0.1% crystal violet solution (Becton Dickinson), and lysed with 10% acetic acid for quantification by colorimetric measurement at 550 nm. Experiments were done in triplicate.

### Cytotoxic Assay

Cells were seeded in 96-well plates (5000 cells/well) in 100 µL of complete medium and incubated overnight for adhesion. Complete medium (100 µL) containing Docetaxel (0 to 100 nM, Sigma-Aldrich) or Doxorubicin (0 to 10 µM, Sigma-Aldrich) was added and cells were incubated for 72 additional hours. Each concentration was performed in 3 replicates. Cytotoxic activity was determined using the XTT cell proliferation Kit (Roche Diagnostics) according to the manufacturer's recommendations.

### Statistical analyses

Data are presented as the Mean ± Standard Error of the Mean. Data analysis was performed using the GraphPad Prism 5 software. P values below 0.05 were considered for the detection of statistically significant differences.

## Supporting Information

Figure S1 **CD146 expression in modified cell lines.** Phenotypic analysis of CD146 expression in MCF-7, SKBR3 cells (forced expression) and CAL51, MDA-MB-231 (down-modulation with shRNAs).
(PPT)

Figure S2 **JAM-A expression in MCF-7 cells transfected with siRNA targeting JAM-A.** Phenotypic analysis of JAM-A expression after siRNA transfection in MCF-7 cells.
(PPT)

Table S1 **Primers used in this study.** Primers used for Q-RT-PCR analysis of EMT markers.
(DOC)
