# Supplementary material for: CD146 Expression in Human Breast Cancer Cell Lines Induces Phenotypic and Functional Changes Observed in Epithelial to Mesenchymal Transition
Source: PLoS One. 2012 Aug 30;7(8):e43752. doi: 10.1371/journal.pone.0043752 (PMC3431364; doi:10.1371/journal.pone.0043752)
Supplement: Figure S1 — CD146 expression in modified cell lines. Phenotypic analysis of CD146 expression in MCF-7, SKBR3 cells (forced expression) and CAL51, MDA-MB-231 (down-modulation with shRNAs). (PPT) [file pone.0043752.s001.ppt]

## Slide 1
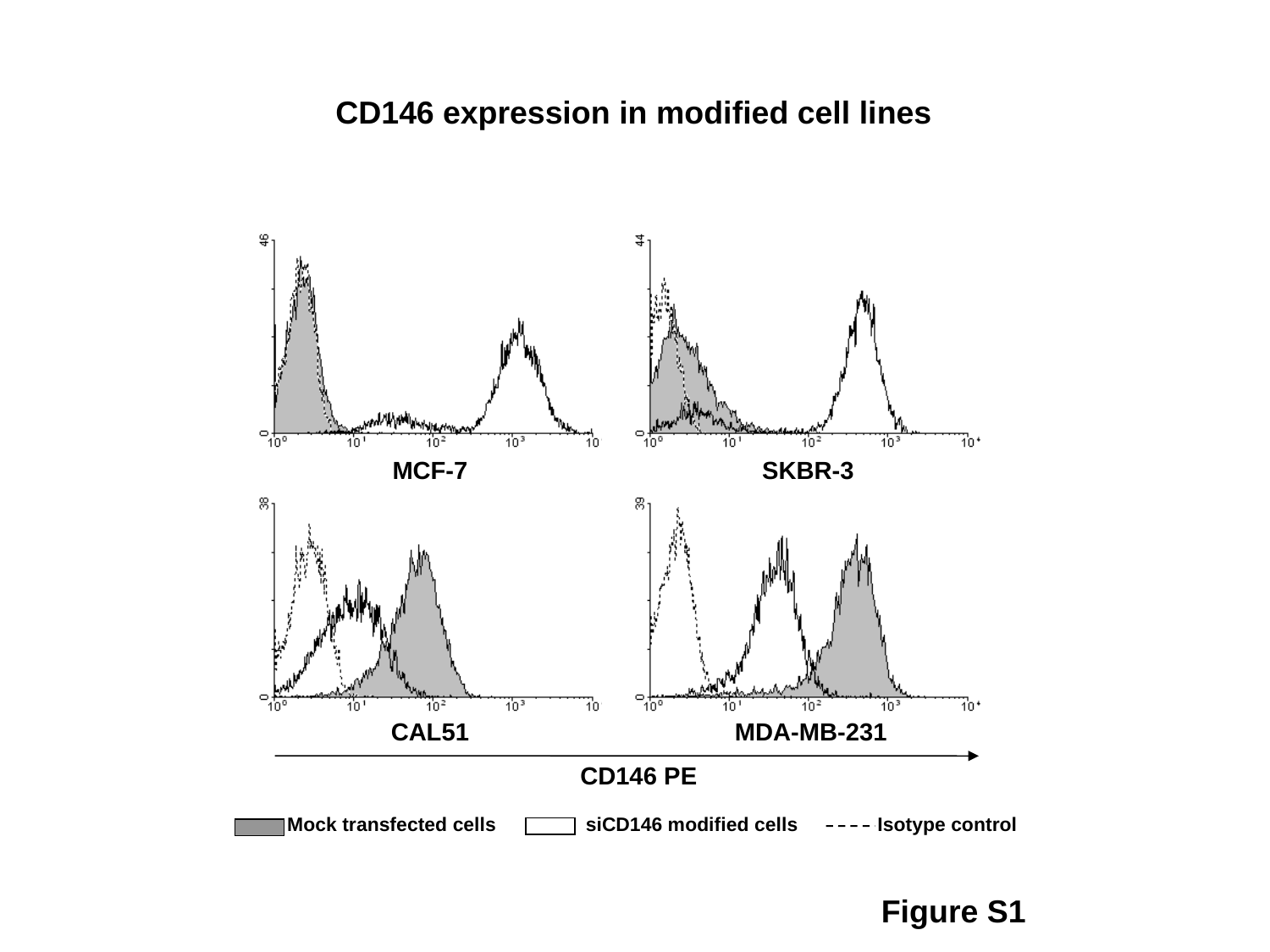

CD146 expression in modified cell lines
MCF-7
SKBR-3
CAL51
MDA-MB-231
CD146 PE
Mock transfected cells
siCD146 modified cells
Isotype control
Figure S1
