# Supplementary material for: CD146 Expression in Human Breast Cancer Cell Lines Induces Phenotypic and Functional Changes Observed in Epithelial to Mesenchymal Transition
Source: PLoS One. 2012 Aug 30;7(8):e43752. doi: 10.1371/journal.pone.0043752 (PMC3431364; doi:10.1371/journal.pone.0043752)
Supplement: Figure S2 — JAM-A expression in MCF-7 cells transfected with siRNA targeting JAM-A. Phenotypic analysis of JAM-A expression after siRNA transfection in MCF-7 cells. (PPT) [file pone.0043752.s002.ppt]

## Slide 1
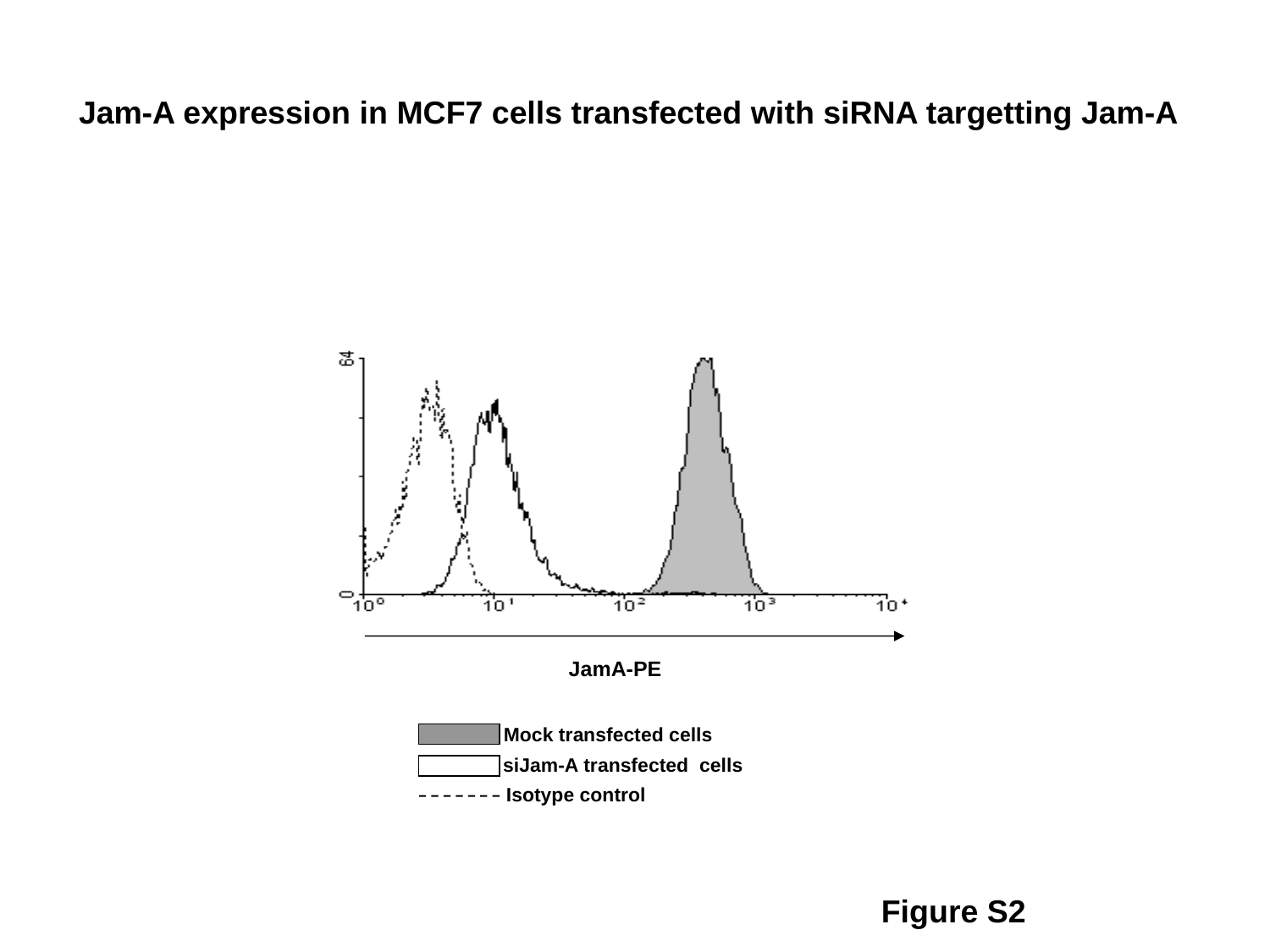

Jam-A expression in MCF7 cells transfected with siRNA targetting Jam-A
JamA-PE
Mock transfected cells
siJam-A transfected cells
Isotype control
Figure S2
