# Supplementary material for: CD146 Expression in Human Breast Cancer Cell Lines Induces Phenotypic and Functional Changes Observed in Epithelial to Mesenchymal Transition
Source: PLoS One. 2012 Aug 30;7(8):e43752. doi: 10.1371/journal.pone.0043752 (PMC3431364; doi:10.1371/journal.pone.0043752)
Supplement: Table S1 — Primers used in this study. Primers used for Q-RT-PCR analysis of EMT markers. (DOC) [file pone.0043752.s003.doc]

**Table S1**

**Primers used in this study**

|  | **Gene Symbol** |  | **Forward** | **Reverse** |
| --- | --- | --- | --- | --- |
| Vimentin | VIM | NM_003380 | aaagtgtggctgccaagaac | agcctcagagaggtcagcaa |
| N-Cadherin | CDH2 | NM_001792 | ctccatgtgccggatagc | cgatttcaccagaagcctctac |
| E-Cadherin | CDH1 | NM_004360 | cccgggacaacgtttattac | gctggctcaagtcaaagtcc |
| Slug | SNAI2 | NM_003068 | tggttgcttcaaggacacat | gttgcagtgagggcaagaa |
| Snail | SNAI1 | NM_005985 | gctgcaggactctaatccaga | atctccggaggtgggatg |
| Twist1 | TWIST1 | NM_000474 | ggagtccgcagtcttacgag | tctggaggacctggtagagg |
| MMP2 | MMP2 | NM_001127891 | ataacctggatgccgtcgt | aggcacccttgaagaagtagc |
| MMP9 | MMP9 | NM_004994 | gaaccaatctcaccgacagg | gccacccgagtgtaaccata |
| CD146 | MCAM | NM_006500 | gggtaccccattcctcaagt | cagtctgggacgactgaatg |
| ER alpha | ESR1 | NM_000125 | cctgatgattggtctcgtctg | ggcacacaaactcctctcc |
| ER beta | ESR2 | NM_001040275 | agagtccctggtgtgaagcaag | gacagcgcagaagrgagcatc |
| PR | PGR | NM_000926 | tgacacctccagttctttgc | aacaccattaagctcatccaag |
| EGFR | EGFR | NM_005228 | catgtcgatggacttccaga | gggacagcttccatcacact |
| Her2 | ERBB2 | NM_001005862 | gggaaacctggaactcaccta | ccctgcacctcctggata |
| Her3 | ERBB3 | NM_001005915 | cacattgccgcactctcc | cacgaggacatagcctgtca |
| Her4 | ERBB4 | NM_001042599 | ttccactttcaaacaacatgcta | cagaatgaagagcccacca |
| beta actine | ACTB | NM_001101 | gagcgcggctacagctt | tccttaatgtcacgcacgattt |
